# Prevalence, Knowledge, Attitude, and Predictors of Waterpipe Smoking among School Adolescents in Saudi Arabia

**DOI:** 10.1155/2022/1902829

**Published:** 2022-09-30

**Authors:** Rashad Alsanosy

**Affiliations:** College of Medicine, Jazan University, Jazan, Saudi Arabia

## Abstract

This cross-sectional study was designed to investigate the prevalence, knowledge, attitude, and predictors of waterpipe (WP) smoking among intermediate and secondary school adolescents in the Kingdom of Saudi Arabia (KSA). A self-administered anonymous questionnaire was used to collect data on demography, WP smoking status and patterns, the Arabic version of the Global Youth Tobacco Survey tool, and instruments to assess knowledge and attitude towards WP smoking. The Patient Health Questionnaire (PHQ-9) was also used. Descriptive and inferential statistical techniques were used. Modeling of WP smoking behavior was conducted using logistic regression. A total of 639 male students participated in this study. The prevalence of current WP and cigarette smokers were 17.7% and 14.6%, respectively. Out of the total population, 47.8% of students have the misconception that WP smoking is less harmful than cigarettes. A significant association (*P* < 0.05) of some demographic factors (age, school stage, residence, and parents' educational level) on WP smoking status was observed. Pleasure, socializing, and happiness represented the primary motives for initiating WP smoking. The majority of students had misconceptions about WP's health effects. More than 50% believed that smoking WP could ease anxiety, cause less harm, and has less addictive properties compared to cigarettes. Modeling suggested that the most significant predictors of WP smoking were cigarette smoking, depression, and the attitude index. Current findings warrant further research and official health programs to promote educational initiatives regarding WP smoking.

## 1. Introduction

Waterpipe (WP), also known as shisha, argileh, narghile, and hookah, tobacco smoking has become a global concern due to its rapid integration into all communities, particularly adolescents and younger populations. WP smoking is responsible for any adverse health problems due to the public's common misperceptions, particularly among adolescents and young adults, as this tobacco product is of low or no harm [1–4]. The absence of health warning labels and their availability in so many different tempting flavors have strengthened such incorrect beliefs about WP in the minds of many adolescents [5]. This situation is worsened by the pleasurable cognitive picture of having fun and relaxing usually associated with the social context of WP smoking [6–8], which increases the chances of addictive WP smoking and nicotine dependence and subsequent complications when quitting smoking. Strictly speaking, many studies have confirmed that WP smoking produces a remarkably elevated group of toxicants compared to cigarette smoking. As with any other form of tobacco (e.g., cigarette, e-cigarette, cigar, and smokeless tobacco), WP smoking poses a severe threat to pulmonary function and can cause lung cancer and cardiovascular diseases [9, 10]. Combined WP and cigarette smoking were reported to be a key correlate of depression among the Iranian population [11].

The habit of WP smoking was highly prevalent among adolescents and students. Essentially, many of those adolescent/student subpopulations were either nonsmokers (3) or combination cigarette and WP smokers [1, 12, 13]. In their study, Jawad et al. [14] concluded that WP smoking was the most prevalent and most commonly smoked tobacco form among 6^th^ and 7^th^-grade Lebanese students; this prevalence was three times higher (22.1%) than that of current cigarette smokers (7.4%) [14]. While in Jordan, it seems that one-half [15] to one-third of the adolescent subpopulation were dual smokers; the habitual prevalence of WP among Jordanian adolescents was found to be double that of cigarette smoking [16]. Similarly, this habit was twice as prevalent as cigarette smoking among Turkish adolescents [17]. In contrast, the perceived health harms of a wide range of tobacco products were investigated among younger subpopulations in the United States. It was found that WP smoking was the second most commonly prevalent tobacco product [18], and from 2011-2012, WP current smokers jumped remarkably among high school students overall from 4.1% to 5.4% (28). The annual prevalence curve of WP smoking habits for the twelfth graders in 2010, 2011, 2012, and 2013 was 17.1%, 18.5%, 18.3%, and 21.4%, respectively, demonstrating rapid tremendous increases. In Pakistan, this habit was widespread among 39% of adolescent students [19].

Previous research was regarding students' attitudes and understanding about WP. It has been established that the male gender and higher middle-income level are connected with waterpipe smoking among students in Jordan. Most pupils had false ideas about waterpipe danger, according to a cross-sectional study carried out in five Mediterranean nations. These data demonstrate the necessity of raising student knowledge of the negative impacts of WP [20, 21]. The prevalence and characteristics of WP smoking are among secondary [5, 22], high school [3, 4, 23], and university [1, 6, 24–26] students in various regions of KSA. However, we did not find any study on both intermediate and secondary school students together in the Jazan region. Therefore, in light of the fact that WP smoking as a habit is becoming a widespread global phenomenon, particularly among adolescents, this paper investigates for the first time the prevalence, knowledge, attitude, and predictors of WP smoking among intermediate and secondary school adolescents in KSA.

## 2. Materials and Methods

### 2.1. Study Design

A quantitative and cross-sectional design using a self-administered survey was conducted to assess the prevalence, knowledge, attitude, and predictors of WP smoking among school adolescents in KSA.

### 2.2. Setting and Sampling

The study was conducted in Jazan administrative district in southwest KSA. According to the local Directorate of Education Jazan, the total population enrolled in schools was approximately 150,000 students from rural and urban areas. The current study data were collected from thirty intermediate and higher secondary schools from five subdistricts (Sabya, Baish, Al-Arabi, Faifa, and Aldera) of the Jazan administrative district. The Raosoft sample size calculator was used to compute the sample size [27]. The sample size was calculated based on a previously reported prevalence [26], 5% type-I error, and 20% type-II error. An additional 20% increase was included as the probable nonresponse rate resulting in a total sample size of 767 students. The sampling technique and randomization were performed and fractioned based on the distribution of the schools. Data were collected proportionally to the number of enrolled students in selected subdistricts and schools. Student registration numbers were used for the randomization process in the respective schools. Data were collected from September 2021 to January 2022.

### 2.3. Study Variables and Procedures

The self-administered questionnaire was distributed anonymously to gather the required information. Different terms are used for WP in the Saudi community. In this study, we used shisha as the local Arabic term.

#### 2.3.1. Demography, Prevalence, and Pattern of Use

Socio-demographic characteristics, including age, student's grade, residence type, monthly income, and parental educational status, were obtained. Prevalence and pattern of WP smoking were determined using the validated Arab version of the Global Youth Tobacco Survey 2001 (GYTS) [5]. Questions used included assessing the frequency of smoking, age at initiation, place of smoking, time spent in smoking sessions, costs, smoking of WP among close friends and family members, primary motives for WP smoking, and quitting attempts in terms of frequency in the last year.

#### 2.3.2. Knowledge and Attitude

Knowledge and attitudes towards WP were assessed. Questions regarding knowledge were answered as correct, incorrect replies, and “do not know.” Attitude questions were set as a Likert scale with five degrees of agreement with the respective items.

#### 2.3.3. Patient Health Questionnaire (PHQ-9)

Depression was measured using the Patient Health Questionnaire (PHQ-9), a nine-item instrument based on the DSM-IV criteria for a major depressive episode. This instrument asks the respondents to indicate the frequency of various symptoms over the past two weeks. Following the standard algorithms for interpreting the PHQ-9, we categorized students as screening positive for major depression, depression, and other depressive categories. This screening tool has been validated and found to be highly correlated with diagnoses made by mental health professionals and other depression assessment tools in various populations [28, 29]. We used the Arabic version of PHQ in this research, which has been validated and evaluated in primary care settings in Arab nations [5].

### 2.4. Data Collection, Reliability, and Validity

Schools were primarily visited to provide orientation sessions to teachers and students regarding study objectives and data collection processes. Researchers were very specific about the location of data collection and questionnaire administration. A pilot study was conducted on 50 students to assess the questionnaire's Arabic translation, reliability, and validity. Cronbach's alphas (the reliability coefficient) for the instruments measuring knowledge, attitude, GYTS, and PHQ-9 selected items were found to be more than 0.70 [30]. The content validities for the instruments of knowledge, attitude, GYTS, and PHQ-9 were established using the reverse translation by professional English editors. Principal component analysis was used to ensure the unidimensionality of the instruments in this study. Explained variances of knowledge, attitude, GYTS, and PHQ-9 were observed to be more than 70%.

### 2.5. Ethical Approval

A personal approach was taken by giving the male students a cover letter with a Jazan University header accompanied by an ethical approval and consent form directed to their parents/legal guardians. This study was approved by the Committee from the Education Directorate, Jazan, KSA.

### 2.6. Data Analysis

Data were entered, processed, and analysed using SPSS version 26.0 (SPSS Inc., Chicago, IL, USA). Missing and incomplete data were removed, not more than 1%. The overall response rate was 92.5%. All areas obtained a response rate of more than 90%. For categorical data, frequencies, proportions, and percentages were employed for the expression of the results. Odds ratios and 95% confidence intervals were estimated whenever appropriate. Chi-squared test for proportions was used to test the significance of comparisons. The results were presented using means, standard deviations, and medians for continuous data, and student-*t*, Mann–Whitney, and Kruskal–Wallis tests of significance were used for comparisons. Scores of the PHQ-9 were classified using the proposed algorithm into any depression, major depression, and depressive disorders [31]. Logistic regression analysis (ENTER Method) was used to determine the predictors of WP smoking behavior. Odds ratios with 95% confidence intervals were calculated for each predictor. Because there are no agreed-upon measures for R^2^ contributions to date in logistic regression, we analysed the data to obtain the Nagelkerke R2 statistic [32], which compares the null model and fitted model likelihood functions as a proportion of the maximum possible R^2^ value. Binary codes were applied to gender (1 = male, 0 = female), current or former cigarette smoking (1 = yes, 0 = no), and depressive disorders (1 = present, 0 = none) in the generation of regression models. The probability levels used for inclusion and removal in the regression model were 0.05 and 0.10, respectively. Statistical significance was set at 0.05.

## 3. Results

### 3.1. Socio-Demographic Characteristics and Prevalence

A total of 639 male students were included in this study (Table 1). The proportions of participants whose ages ranged from 13 to 15 years and 16 to 18 years were 46.2% and 42.4%, respectively. Proportions of students from intermediate and secondary schools were 50.7% and 49.3%, respectively. It should also be noted that most of the respondents were from urban areas and had a monthly income of SAR300 or more (1 USD = 3.77SAR). Chi-squared tests were used to determine whether frequency counts were distributed identically across different populations of age, school stage, residence, and parents' educational level. These were then cross-tabulated with WP smoking status. Chi-squared tests with *P* values of less than 0.05 indicated a significant association with demographic factors, as shown in Table 1. The prevalence of current WP and cigarette smoking were 17.7% and 14.6%, respectively. This study included WP smoking (111 smokers and 510 nonsmokers) and cigarette smoking (89 smokers and 531 nonsmokers). A 2 × 2 chi-squared test of independence was used to determine if WP smoking was dependent on cigarette smoking. Given *α* = 0.05, the results suggest dependency, *χ*^2^(1,*N* = 639) = 284.02, *P* < 0.001.

### 3.2. Pattern of Use and Motives

The WP smoking patterns of participants are depicted in Table 2. The majority of the respondents (>50.0%) started their WP and cigarette smoking at 13 to 15 years. Daily, weekly, and monthly WP users represent 26.7%, 34.4%, and 38.9% of the study participants, respectively. Table 2 displays the stated primary motives concerning the current smoking status. Students indicated that their primary motives for starting WP smoking were pleasure and happiness. Moreover, 41.8% of the students smoked waterpipe with their friends.

### 3.3. Adolescents' Knowledge and Attitudes and PHQ-9

The correct responses regarding the health hazards of WP smoking concerning the current smoking status of the included adolescents are displayed in Table 3. Out of the total population, 47.8% of students have the misconception that WP smoking is less harmful than cigarettes. 59.2% believed that harmful substances are purified through water filtration in the WP, and 65.9% stated that WP does not have addictive properties. A chi-squared test was used to analyze the distribution of the responses regarding knowledge of the health effects of WP smoking among the included participants concerning their current smoking status. As shown in Table 3, findings revealed statistical significance (chi-squared value = 187), except for knowledge regarding the harmlessness of the amount of nicotine in the WP.  Table 4 depicts the answers regarding attitudes towards WP smoking among school adolescents. The mean attitude index for WP smokers (*n* = 111) and non-WP smokers (*n* = 506) was compared using the Mann–-Whitney *U* test at = 0.01. The results suggest that average WP smokers' scores (*M* = 2.079, SD = 0.63) are significantly lower than average non-WP smokers' scores (*M* = 2.803, SD = 0.7131), *U* = 12899, *P* < 0.001. When looking at the individual items of the attitude questions, we observed that of the included adolescents, the majority agreed that WP smoking is more socially acceptable than cigarettes and represents an excellent opportunity for gathering with friends and family; this finding was considerably more frequent among WP smokers. Additionally, more than 50% of participants believed that WP smoking could ease anxiety, cause less harm, and has less addictive properties compared to cigarettes.

The overall mean for PHQ-9 instruments is 7.76 ± 2.63. According to current smoking status, mental health screening (PHQ-9) results are expressed as percentages. WP smokers show a prevalence of 69.9%, 13.3%, and 16.9% for other depression, major depression, and any depression, respectively. A 2 × 3 chi-squared test of independence showed a significant association of PHQ-9 classification of severity of depression with WP smoking (*χ*2 = 9.175, *P* value <0.05).

### 3.4. Predictors of Waterpipe Use

WP smoking behavior was modeled using logistic regression based on prospective predictors. This includes age, school stage, cigarette smoking, education, monthly expenses, depression status, and attitude index. A preliminary univariate analysis was conducted to identify potential risk factors, followed by a binomial multivariate logistic regression analysis. The results are shown in Table 5. In this model, 60.9% (Nagelkerke R^2^) of WP smoking status could be explained by including these predictors. The modeling suggests that the most significant independent predictors of WP smoking were cigarette smoking (OR = 0.018, *P* < 0.001), depression (OR = 0.03980, *P* = 0.036), and attitude index (OR = 3.028, *P* = 0.01). This result explained the positive relationship between WP smoking and the attitude score. However, as explained by the regression model, monthly expenses, education, housing, school, and age were insignificant predictors for WP smoking.

## 4. Discussion

Waterpipe smoking is a major public health issue. WP and cigarette smoking have recently seen an unprecedented rise in popularity worldwide. This study is one of the few from the region that highlights the prevalence and predictors of WP smoking usage among intermediate and secondary school male adolescents and their knowledge, attitude, and psychological health. The current findings demonstrated that the prevalence of WP smoking amongst Saudi adolescents from the Jazan Region is 17.7%, and WP smoking is dependent on cigarette smoking (*P* < 0.001). 80.9% of current smokers were also WP smokers. These numbers are higher than those reported in previous studies conducted on students from various regions of KSA [22, 26, 33]. This study's higher figures could explain a large proportion of the Jizani population chew khat [34], and the region is less urbanized. In different communities, tobacco use among khat chewers is reported as ordinary or only during khat chewing. Khat chewing may encourage diverse types of tobacco smoking, the commencement and maintenance of tobacco smoking, and prompt cessation relapses. Increased incidence of tobacco smoking was associated with psychophysical and behavioral factors [35]. WP smoking is being used increasingly amongst young adults in the Middle East [36]. This increase could be explained by the enhanced commercial sector of WP devices, accessories, and Muassel in Egypt and Syria. Muassel is a syrupy tobacco mix containing molasses and vegetable glycerol as moisturizers with specific flavors added to it. Muassel is moist and pliable, making it easier to use than other WP tobaccos, and it has a pleasant taste and aroma, thus recruiting new tobacco users [37, 38]. Many factors have also led to the recent widespread usage of WPs, including lowered danger perception and a vibrant café and restaurant culture [39, 40].

The students participating in this study were asked about the primary motive for smoking WP, and the choices were family problems, experience with family, experience with friends, or the search for pleasure. Searching for fun and experience with friends are the major motives reported in this study. These motives could explain the strong association of WP smoking behaviors among the included students. WP smoking in the Arabic world represents a unique cultural and traditional phenomenon, and it is used as a form of relaxation, hospitality, and socialization [41, 42].

The rate found in this study is lower than rates seen in previous studies [43, 44], which indicates that study participants were more aware of the negative consequences of WP. According to studies, most students believed that using marijuana as a substitute for smoking cigarettes was less dangerous, less addictive, and more acceptable in society [43]. WP smoking is common in KSA, and it is linked to a lack of knowledge about the risks and how to quit [22, 26, 45]. In this study, students had the misconception that WP smoking is less harmful than cigarettes, and 59.2% of them believed that harmful substances are purified through water filtration in a WP, while 65.9% stated that WP does not have addictive properties. These opinions regarding the limited hazard of WP smoking may help clarify why some adolescents who do not smoke cigarettes are enthusiastic about engaging in WP use. The current results suggest that WP smoker scores are significantly less than average nonsmokers' scores on their attitude index, supporting previous findings [44]. When looking at the individual attitudes question items, we observed that most of the included adolescents agreed that WP smoking is more socially acceptable than cigarettes and represents an excellent opportunity to gather with friends and family, which was considerably more frequent among WP smokers. Additionally, more than 50% believed that WP smoking could ease anxiety, cause less harm, and has less addictive properties compared to cigarettes. According to research conducted at Jordan's top colleges, 44.4% believe WP smoking is more socially acceptable than cigarette smoking, 34.2% believe both are equally socially acceptable, and just 21.5% believe WP smoking is less socially acceptable [12].

The multivariate logistic regression used age, school stage, cigarette smoking, education, monthly expenses, depression status, and attitude index. In this model, 60.9% of WP smoking status could be explained by including these predictors, with cigarette smoking, depression, and attitude index being the most significant ones (*P* < 0.05). A significant association between the PHQ-9 classification of depression and WP smoking was observed in this study. Previous studies have linked smoking with some antidepressant effects, which explains why cigarette smoking is much more common among depressed patients [46]. A US study confirmed that long-term depressive disorders were more frequent among smokers than nonsmokers and described that smokers who reported at least one incident of major depression were less likely to succeed in smoking cessation programs than smokers without depression [47, 48]. Current findings also suggested that the cumulative attitude index correlates positively with WP smoking status amongst Jazani adolescents. The attitude was measured based on the students' perceptions of individual and parental acceptability, socializing roles, health effects, and addictiveness. A previous study suggested that WP users were aware of the health hazards of WP smoking, but they perceived it as less harmful, less addictive, and more socially acceptable than cigarette smoking and were confident in their ability to quit [49]. Age was not found to be significantly associated with WP. This may result from the fact that the ages of the school students in this study are similar in their values. Also, the presence of other factors in the used model led to the neutralization of the role of age in the use of WP.

### 4.1. Limitations

The results of this study are based on a cross-sectional design, so causal inferences cannot be made. A longitudinal study is required to understand the causative roots of the variables included in this study. Another limitation is the exclusion of female adolescents in the population. Another limitation is the lack of information on the smoking behaviors of nonrespondents (particularly females) and those adolescents not in school. It is possible that nonrespondents had higher smoking prevalence than those surveyed, leading to a subsequent underestimation of smoking prevalence rates. Laboratory validation of psychometric instruments was also a limitation. Additionally, the lack of standardized interviews in data collection is a limitation as these are considered more potent than self-reporting methods. The generalization of the findings can also be affected by the civilization status and geographical location of the Jazan region, which is considered less urbanized. The regression model used in this study could not explain all the variability in WP smoking behaviors amongst adolescents, and additional predictors are required. Moreover, khat as a confounding factor should have been included in the current study.

## 5. Conclusion

This study highlighted the prevalence and risk factors of WP smoking among Saudi adolescents. The region showed a high prevalence of WP smoking, dependent on cigarette smoking. We noticed an alarming trend towards a rising use of WP smoking linked with substantial certainty that it is less unsafe than cigarettes. Modeling of WP use was performed using logistic regression and its prospective predictors. Models suggest that the most significant independent predictors of WP smoking were cigarette smoking, depression, and attitude index. Additionally, it is essential to focus on the levels of depression among current adolescent smokers. Counseling and preventive psychiatric services should be an essential component of the clinical facilities caring for school students in KSA.

## Figures and Tables

**Table 1:** Socio-demographics of the included school adolescents (*n* = 639) and their relationship to current smoking behaviors

	Current smoking status (no. %)			Chi-square test value (*P* value)
	Waterpipe	Nonsmoker	Total	
Age (yrs)				**27.271 (0.001)**
13–15	32 (5.2)	258 (41.8)	290 (47)	
16–18	56 (9.1)	210 (34.0)	266 (43.1)	
Over 18	23 (3.7)	38 (6.2)	61 (9.9)	
Stage				**20.915 (0.001)**
Intermediate school	34 (5.6)	276 (45.1)	310 (50.7)	
Secondary school	76 (12.4)	226 (36.9)	302 (49.3)	
Residence				0.305 (0.581)
Urban	21 (3.4)	109 (17.6)	130 (21.0)	
Rural	89 (14.4)	399 (64.6)	488 (79.0)	
Monthly expenditure				**8.72 (0.003)**
Less than 300 SAR	51 (8.4)	310 (51.0)	361 (59.4)	
More than 300 SAR	58 (9.5)	189 (31.1)	247 (31.1)	
Father's education level				1.321 (0.724)
Uneducated	25 (4.1)	124 (20.4)	149 (24.5)	
Elementary	31(5.1)	150 (24.7)	181 (29.8)	
Senior	23(3.8)	82 (13.5)	105 (17.3)	
University and above	31 (5.1)	142 (23.4)	173 (28.5)	
Mother's education level				4.737 (0.192)
Uneducated	42 (7.0)	180 (29.9)	222 (36.9)	
Elementary	28 (4.7)	144 (23.9)	172 (28.6)	
Senior	13 (2.2)	81 (13.5)	94 (15.6)	
University and above	28 (4.7)	86 (14.3)	114 (18.9)	
Cigarette smoking				**284.02 (0.001)**
Yes	72 (11.7)	17 (2.7)	89 (14.4)	
No	38 (6.1)	493 (79.5)	531 (85.6)	

**Table 2:** Waterpipe smoking patterns of participants.

	Number	Percentage
Age at first use of waterpipe^*∗*^		
13 to 15	80	57.1
16 to 18	40	28.6
More than	20	14.3
Age at first use of cigarettes		
Less than 13	1	0.9
13 to 15	66	56.9
16 to 18	32	27.6
More than	17	14.7
Frequency of waterpipe smoking		
Daily	35	26.7
Weekly	45	34.4
Monthly	51	38.9
Place of waterpipe smoking^*∗*^		
Home	27	20.1
Coffee shop	51	38.1
With friends (break)	56	41.8
Primary motives for smoking^*∗*^		
Pleasure and happiness	63	48.8
Experiencing with friends	45	34.9
Experiencing with family	6	4.70
Emotional and family problems	15	11.6

^*∗*^ *P* < 0.05; chi-squared test.

**Table 3:** Knowledge regarding the health effects of waterpipe (Shisha) smoking among included participants in relation to their current smoking status.

Knowledge items	Correct response (no., %)			Chi-squared (*P* value)
	Waterpipe smoking			
	Yes	No	Total	
Shisha smoking is less harmful compared to cigarettes (false)	45 (7.2)	201 (32.2)	246 (39.4)	47.796 (0.001)
Shisha is purified of harmful substances after passing through a water filter (false)	33 (5.3)	89 (14.4)	122 (19.7)	66.303 (0.001)
Shisha contains a harmless amount of nicotine and tar (false)	36 (5.8)	151 (24.4)	187 (30.2)	187 (30.2)
Shisha smoking does not irritate the bronchi as it contains natural flavours (false)	45 (7.3)	185 (29.9)	230 (37.2)	64.328 (0.001)
Shisha smoking is easier to quit and is not addictive (false)	47 (7.6)	207 (33.4)	254 (41.0)	107.456 (0.001)
Shisha smoking does not cause lung cancer as opposed to cigarettes (false)	53 (8.5)	200 (32.2)	253 (40.7)	39.819 (0.001)
Shisha smoking causes damage to the respiratory system (true)	71 (11.5)	225 (36.3)	296 (47.7)	38.481 (0.001)
Shisha smoking may transmit hepatitis infection (true)	56 (9.0)	146 (23.6)	202 (32.6)	58.907 (0.001)
Shisha smoking increases the risk of pharyngeal cancer (true)	55 (8.9)	163 (26.2)	218 (35.1)	53.599 (0.001)
*Helicobacter* infections that cause gastric ulcers could be transmitted through shisha smoking (true)	49 (7.9)	116 (18.7)	165 (26.7)	45.855 (0.001)
Shisha smoking does not cause cardiovascular diseases, such as coronary heart disease, compared to other forms of smoking (false)	37 (6.0)	133 (21.5)	170 (27.5)	49.285 (0.001)
Shisha smoking may transmit tuberculosis and leprosy (true)	46 (7.5)	104 (16.9)	150 (24.3)	43.512 (0.001)

**Table 4:** Attitudes towards waterpipe smoking among secondary school students.

Questions of attitudes	Waterpipe smoker (WPS)/nonsmokers (WPNS) agreement (N)												
	WPS	WPNS	WPS	WPNS	WPS	WPNS	WPS	WPNS	WPS	WPNS	Attitude index^*∗*^		
	Strongly agree		Agree		Do not know		Disagree		Strongly disagree		WP smoker	WP nonsmoker	Test statistic^*∗∗*^ (*P* value)
1. Shisha smoking is acceptable by society compared to cigarettes.	29	46	21	51	29	260	12	67	17	84	2.079 ± 0.63	2.803 ± 0.7131	12899 (0.001)
2. Shisha smoking represents a good opportunity to meet friends and family.	22	18	16	32	22	186	26	94	22	178			
3. My parents would not object to my smoking of shisha compared to cigarettes	18	18	13	25	27	223	23	78	26	162			
4. My parents would allow me to smoke shisha at home but not cigarettes	11	8	9	12	27	176	25	86	35	228			
5. Shisha smoking is a sign of maturity	15	18	16	17	29	200	19	77	28	196			
6. Smoking shisha relieves stress and tension	29	23	17	22	24	227	12	79	24	154			
7. If I have to smoke, I would use shisha because it is less harmful and less addictive	26	34	13	35	26	252	17	69	22	114			
8. Shisha smokers have more friends than nonsmokers	32	28	20	43	21	266	11	63	22	107			
9. Women smoking shisha are not as old as those smoking cigarettes	25	27	11	23	38	298	12	57	20	99			
10. Movie stars smoking shisha are more acceptable than those smoking cigarettes	21	25	11	27	39	278	16	67	19	106			

^*∗∗*^Mann–Whitney U; WPS: waterpipe smokers; WPNS: waterpipe nonsmoker.

**Table 5:** Modeling of waterpipe smoking using logistic regression.

Predictors	B	*P* value	OR	95% C.I. for OR	
				Lower	Upper
Age (reference group is over 18)		0.51			
13 to 15	−0.16	0.84	0.85	0.17	4.17
16 to 18	−0.59	0.33	0.55	0.16	1.845
School (reference group is senior school)	−0.09	0.87	0.91	0.29	2.86
Housing (reference group is village)	−0.94	0.06	0.38	0.141	1.07
Education (reference group is special education)	1.36	0.05	3.89	0.973	15.60
Expenses (reference group is more than 300 SR)	0.12	0.73	1.13	0.54	2.37
Cigarettes (reference group is no)	−3.9	**0.01**	0.01	0.00	0.04
Depressive disorders (yes/no)	1.10	**0.01**	3.02	1.77	5.17
Attitude index	0.92	**0.03**	2.51	1.06	5.95
Constant	−1.66	0.10	0.19		

• Modeling of waterpipe use was performed using logistic regression and its prospective predictors. These included age, school stage, cigarette smoking, kind of education, monthly expenses, depression status, and attitude index. • Hosmer and Lemeshow goodness of fit test *χ*2 = 5.532, *P* = 0.699; −2 log likelihood ratio = 205.097. • B: regression coefficient; OR: odds ratio; C.I.: confidence intervals.

## Data Availability

The datasets used and/or analysed during the current study are available from the author upon reasonable request.
